# Supplementary material for: Unveiling the Bioactive Potential of the Invasive Jellyfish Phyllorhiza punctata Through Integrative Transcriptomic and Proteomic Analyses
Source: Biomolecules. 2025 Aug 4;15(8):1121. doi: 10.3390/biom15081121 (PMC12383608; doi:10.3390/biom15081121)
Supplement: Supplementary file 1 [file biomolecules-15-01121-s001.zip › Suplementary Material/FileS3/PEPFOLD4/XP_020913125_428/clusters.html]

 PEP-FOLD clusters


Download archive of all models  here   

|  |  |  |
| --- | --- | --- |
| # | file | sOPEP |
| # cluster 1 - Size: 94 |  |  |
|  | XP\_020913125\_428-model1 | -26.8959 |
|  | XP\_020913125\_428-model1.1 | -26.8426 |
|  | XP\_020913125\_428-model1.2 | -26.6714 |
|  | XP\_020913125\_428-model1.3 | -26.668 |
|  | XP\_020913125\_428-model1.4 | -26.6567 |
|  | XP\_020913125\_428-model1.5 | -26.6434 |
|  | XP\_020913125\_428-model1.6 | -26.5838 |
|  | XP\_020913125\_428-model1.7 | -26.5734 |
|  | XP\_020913125\_428-model1.8 | -26.5525 |
|  | XP\_020913125\_428-model1.9 | -26.5294 |
|  | XP\_020913125\_428-model1.10 | -26.5151 |
|  | XP\_020913125\_428-model1.11 | -26.5039 |
|  | XP\_020913125\_428-model1.12 | -26.5009 |
|  | XP\_020913125\_428-model1.13 | -26.4828 |
|  | XP\_020913125\_428-model1.14 | -26.4809 |
|  | XP\_020913125\_428-model1.15 | -26.4639 |
|  | XP\_020913125\_428-model1.16 | -26.4636 |
|  | XP\_020913125\_428-model1.17 | -26.4509 |
|  | XP\_020913125\_428-model1.18 | -26.4466 |
|  | XP\_020913125\_428-model1.19 | -26.4448 |
|  | XP\_020913125\_428-model1.20 | -26.4421 |
|  | XP\_020913125\_428-model1.21 | -26.4346 |
|  | XP\_020913125\_428-model1.22 | -26.421 |
|  | XP\_020913125\_428-model1.23 | -26.4208 |
|  | XP\_020913125\_428-model1.24 | -26.4178 |
|  | XP\_020913125\_428-model1.25 | -26.4167 |
|  | XP\_020913125\_428-model1.26 | -26.416 |
|  | XP\_020913125\_428-model1.27 | -26.4117 |
|  | XP\_020913125\_428-model1.28 | -26.4065 |
|  | XP\_020913125\_428-model1.29 | -26.4018 |
|  | XP\_020913125\_428-model1.30 | -26.3913 |
|  | XP\_020913125\_428-model1.31 | -26.3869 |
|  | XP\_020913125\_428-model1.32 | -26.3817 |
|  | XP\_020913125\_428-model1.33 | -26.376 |
|  | XP\_020913125\_428-model1.34 | -26.3746 |
|  | XP\_020913125\_428-model1.35 | -26.3721 |
|  | XP\_020913125\_428-model1.36 | -26.3719 |
|  | XP\_020913125\_428-model1.37 | -26.3686 |
|  | XP\_020913125\_428-model1.38 | -26.3649 |
|  | XP\_020913125\_428-model1.39 | -26.3614 |
|  | XP\_020913125\_428-model1.40 | -26.3594 |
|  | XP\_020913125\_428-model1.41 | -26.3517 |
|  | XP\_020913125\_428-model1.42 | -26.3471 |
|  | XP\_020913125\_428-model1.43 | -26.3417 |
|  | XP\_020913125\_428-model1.44 | -26.3414 |
|  | XP\_020913125\_428-model1.45 | -26.3343 |
|  | XP\_020913125\_428-model1.46 | -26.3216 |
|  | XP\_020913125\_428-model1.47 | -26.3146 |
|  | XP\_020913125\_428-model1.48 | -26.3146 |
|  | XP\_020913125\_428-model1.49 | -26.3008 |
|  | XP\_020913125\_428-model1.50 | -26.2995 |
|  | XP\_020913125\_428-model1.51 | -26.2994 |
|  | XP\_020913125\_428-model1.52 | -26.2991 |
|  | XP\_020913125\_428-model1.53 | -26.2957 |
|  | XP\_020913125\_428-model1.54 | -26.2919 |
|  | XP\_020913125\_428-model1.55 | -26.2771 |
|  | XP\_020913125\_428-model1.56 | -26.2744 |
|  | XP\_020913125\_428-model1.57 | -26.2602 |
|  | XP\_020913125\_428-model1.58 | -26.2532 |
|  | XP\_020913125\_428-model1.59 | -26.2001 |
|  | XP\_020913125\_428-model1.60 | -26.1989 |
|  | XP\_020913125\_428-model1.61 | -26.1845 |
|  | XP\_020913125\_428-model1.62 | -26.1784 |
|  | XP\_020913125\_428-model1.63 | -26.17 |
|  | XP\_020913125\_428-model1.64 | -26.1652 |
|  | XP\_020913125\_428-model1.65 | -26.1638 |
|  | XP\_020913125\_428-model1.66 | -26.1614 |
|  | XP\_020913125\_428-model1.67 | -26.1378 |
|  | XP\_020913125\_428-model1.68 | -26.1313 |
|  | XP\_020913125\_428-model1.69 | -26.1095 |
|  | XP\_020913125\_428-model1.70 | -26.108 |
|  | XP\_020913125\_428-model1.71 | -26.1047 |
|  | XP\_020913125\_428-model1.72 | -26.1045 |
|  | XP\_020913125\_428-model1.73 | -26.0746 |
|  | XP\_020913125\_428-model1.74 | -26.0535 |
|  | XP\_020913125\_428-model1.75 | -26.0512 |
|  | XP\_020913125\_428-model1.76 | -26.0417 |
|  | XP\_020913125\_428-model1.77 | -26.0389 |
|  | XP\_020913125\_428-model1.78 | -26.0389 |
|  | XP\_020913125\_428-model1.79 | -26.0316 |
|  | XP\_020913125\_428-model1.80 | -25.9888 |
|  | XP\_020913125\_428-model1.81 | -25.9655 |
|  | XP\_020913125\_428-model1.82 | -25.9634 |
|  | XP\_020913125\_428-model1.83 | -25.9428 |
|  | XP\_020913125\_428-model1.84 | -25.9389 |
|  | XP\_020913125\_428-model1.85 | -25.912 |
|  | XP\_020913125\_428-model1.86 | -25.8647 |
|  | XP\_020913125\_428-model1.87 | -25.8461 |
|  | XP\_020913125\_428-model1.88 | -25.8212 |
|  | XP\_020913125\_428-model1.89 | -25.7869 |
|  | XP\_020913125\_428-model1.90 | -25.4706 |
|  | XP\_020913125\_428-model1.91 | -25.4478 |
|  | XP\_020913125\_428-model1.92 | -25.4183 |
|  | XP\_020913125\_428-model1.93 | -25.1114 |
| # cluster 2 - Size: 6 |  |  |
|  | XP\_020913125\_428-model2 | -22.1952 |
|  | XP\_020913125\_428-model2.1 | -21.9956 |
|  | XP\_020913125\_428-model2.2 | -21.8685 |
|  | XP\_020913125\_428-model2.3 | -21.7397 |
|  | XP\_020913125\_428-model2.4 | -21.7097 |
|  | XP\_020913125\_428-model2.5 | -21.0944 |
|  |  |  |
